# Permanganate of Potash as a Remedy for Diphtheria

**Published:** 1865-02

**Authors:** Leuis Mackall

**Affiliations:** Georgetown, D.C.


### PERMANGANATE OF POTASH AS A REMEDY FOR DIPHTHERIA.

By LOUIS MACKALL, Jr., M.D., Georgetown, D.C.

Having used for several months past the permanganate of potash as a remedy for diphtheria, and being convinced of its great efficacy, I feel justified in calling the attention of the profession to the use of this agent in this fatal and hitherto unmanageable disease.

After using faithfully all the remedies, both general and local, which have been extolled for the cure of diphtheria, and having seen so little good result from their use, I had lost, in a great measure, all faith in such remedies, and had come to the conclusion that the best treatment was to support the patient with nourishment and the free use of stimulants. On reading, in the January number of the *American Journal*, an article by Dr.

Samuel Jackson on the therapeutical application of a solution of the permanganate of potash and of ozone, it occurred to me that this agent might be beneficial in the treatment of diphtheria. Shortly afterwards I had an opportunity of making a trial of it in a severe case. A young girl about eleven years of age, was seen by me after being sick several days. The tonsils, soft palate, and fauces were covered with an ash-colored deposit; the glands beneath the jaw were much swollen, with frequent pulse and hot skin; she was treated for several days, with chlorate of potash and the tincture of the chloride of iron. Muriatic acid and tincture of iron, in equal parts were applied locally. But finding the disease on the increase, I changed this treatment and used the permanganate of potash both internally and as a local application, the latter in the proportion of ʒj to water Oj. She took a teaspoonful every three hours of the strength of ʒj to water Oiss. On the second day after beginning this treatment, the improvement was very marked, and she speedily recovered. The false membrane was detached and the mucous membrane presented a healthy appearance in three or four days.

Since then I have treated all the cases of diphtheria (some fourteen or fifteen) which I have seen with this agent, and am more and more convinced with every case, that we have in the permanganate a most valuable remedy. Such is my faith in its power to arrest the extension of the pseudo-membranous formation, and to remove it when formed, that I now feel little apprehension in any case if called to see the patient before the disease has extended to the larynx or paralysis has occurred. Indeed, in those almost hopeless cases in which it is evident that the disease has reached the larynx, as shown by suppressed cough and voice with paroxysms of intense dyspnœa, I have seen under its use three children recover. With a considerable experience in the disease I had previously known only one child to recover under similar circumstances. These three cases were all of the most unfavorable character; the membranous formation was abundant; the laryngeal symptoms very distressing. In all of the cases I expressed a very gloomy prognosis, as all similar cases, with the one exception above mentioned, had proved speedily fatal. In these cases I also used emetics, but I think the successful result should be attributed to the permanganate, as I had used emetics in all such cases before without benefit.

When the disease has extended beyond the reach of this remedy locally applied, of course a successful result could not reasonably be expected from its use; but I believe that with this agent we can prevent diphtheria from progressing to a fatal termination, provided the cases can be attended to before the larynx becomes involved.

Its tendency to attack the mucous membrane of the pharynx prior to its extension to the larynx is characteristic of diphtheria, and I feel assured from my experience that if the permanganate of potash is used in this stage, that it will not only control its further development, but will speedily remove all traces of the disease by restoring the mucous membrane of the throat to a healthy state.

The inferences that it is intended should be drawn from the foregoing remarks are: that if diphtheria arises from a specific cause affecting the whole system, then the permanganate of potash may be regarded as the antidote to this poison; or if the fatal tendency is thought to be caused by or to be dependent on the local affection of the throat, then the local affection may be removed and the fatal tendency may be obviated by the use of this remedy.

It may be well to state that I have never seen any unpleasant effect from the use of the permanganate, even when administered to young infants (the solution should be weakened by increasing the quantity of water to Oij to permanganate ʒj in very young children); and I have observed that when locally applied it causes less distress than almost any other remedy.—*Am. Jour. Med. Sciences.*

